# A specialist-led interprofessional weaning program to prevent weaning failure in high-risk patients: the PRiVENT study

**DOI:** 10.1186/s13054-026-06357-3

**Published:** 2026-09-30

**Authors:** Franziska C. Trudzinski, Benjamin Neetz, Biljana Joveš, Axel Kempa, Armin Schneider, Elena Biehler, Thomas Fleischhauer, Janina Schubert-Haack, Teresa Byczkowski, Manuel Feißt, Peter Rosenberger, Jörg Krebs, Simone Britsch, Joachim Szecsenyi, Felix J. F. Herth, the PRiVENT-Study Group, Felix J. F. Herth, Gabriele Iberl, Julia Dorothea Michels-Zetsche, Jana Christina Dahlhoff, Michael Müller, Benjamin Neetz, Franziska Christina Trudzinski, Philipp Höger, Elena Biehler, Thomas Fleischhauer, Gerhard Fuchs, Nicola Litke, Markus Qreini, Joachim Szecsenyi, Michel Wensing, Timm Frerk, Thomas Grobe, Janina Schubert-Haack, Anja Klingenberg, Teresa Byczkowski, Manuel Feißt, Axel Kempa, Biljana Joves, Andreas Rheinhold, Ahmed Ehab, Claus Neurohr, Alessandro Ghiani, Nina Lutz, Swenja Walcher, Konstantinos Tsitouras, Joanna Paderewska, Selina Briese, Armin Schneider, Christoph Rauch, Patrick Gehrig, Joachim Sugg, Susanne Hirschmann, Simone Britsch, Christa Straub, Claude Jabbour, Michael Hahn, Jörg Krebs, Peter-Tobias Graf, Petra Denzer, Uta Merle, Monica Boxberger, Mascha Fiedler-Kalenka, Guido Hundt, Jens Regula, Thushira Weerawarna, Miriane Bomeken, Lisa Amega, Shumallah Basit, Sebastian Stier, Matthias Körner, Jens Müller, Sergej Markin, Ute Oltmanns, Oliver Gorgs, Mark Hackbarth, Sebastian Münz, Dominik Scharpf, Thomas J. Dengler, Mathias Borst, Brigitte Mayer, Wolfgang Reikow, Markus Kredel, Patrick Keppeler, Konstantin Frey, Holger Wolff, Florian Seidlitz, Stefanie Bientzle, Boris Nohé, Sebastian Allgäuer, Alexej Schöpp, Jörg Winckelmann, Christoph Schlegel, Imke Hübner, Andrzej Kuzniar, Helene Häberle, Alice Bernard, Reimer Riessen, Benjamin Schempf, Ingo Rebenschütz, Andreas Straub, Marc Kollum, Markus Winter, Paul Hartveg, Andreas Junginger, Thomas Abt, Mathias Vogel, Ralf Völker, Thomas Wiesmann

**Affiliations:** 1 Department of Pneumology and Critical Care, Thoraxklinik Heidelberg gGmbH, Heidelberg, Germany; 2 Translational Lung Research Center Heidelberg (TLRC-H), German Center for Lung Research (DZL), Heidelberg, Germany; 3 Department of Pneumology and Critical Care, SLK-Klinik Löwenstein, Löwenstein, Germany; 4 Department of Pneumology and Respiratory Medicine, RBK Lungenzentrum Stuttgart at Robert-Bosch-Krankenhaus, Stuttgart, Germany; 5 Department of Anesthesia and Intensive Care Medicine Waldburg-Zeil Kliniken, Wangen im Allgäu, Germany; 6 Department of General Practice and Health Services Research, University Hospital Heidelberg, Heidelberg, Germany; 7 aQua Institute for Applied Quality Improvement and Research in Health Care, Göttingen, Germany; 8 Institute of Medical Biometry, University of Heidelberg, Heidelberg, Germany; 9 Department of Anesthesiology and Intensive Care Medicine, University Hospital Tübingen, Hoppe-Seyler-Straße 3, 72076 Tübingen, Germany; 10 Department of Anesthesiology and Critical Care Medicine, University Medical Centre Mannheim, Medical Faculty Mannheim, University of Heidelberg, Theodor-Kutzer-Ufer 1-3, 68165 Mannheim, Germany; 11 Department of Cardiology, Angiology, Haemostaseology and Medical Intensive Care, Centre for Acute Cardiovascular Medicine Mannheim (ZKAM), University Medical Centre Mannheim, Medical Faculty Mannheim, Heidelberg University, 69117 Mannheim, Germany; 12 German Centre for Cardiovascular Research (DZHK), Partner Site Heidelberg/Mannheim, 13092 Mannheim, Germany; 13 European Center for Angioscience (ECAS), Medical Faculty Mannheim, Heidelberg University, 68167 Mannheim, Germany

**Keywords:** Mechanical ventilation, Weaning, Prolonged weaning, Critical care, Interprofessional care, Implementation science

## Abstract

**Purpose:**

Effective strategies to prevent weaning failure and prolonged invasive mechanical ventilation (IMV) in patients at high risk of weaning failure are limited. We evaluated a structured, specialist-led interprofessional weaning program.

**Methods:**

In this prospective, multicenter, non-randomized interventional study, mechanically ventilated patients were screened 96 h to 11 days after intubation. Patients meeting predefined high-risk criteria received a specialist-led interprofessional weaning program, while a parallel control group was derived from health insurance claims data. The primary outcome was successful weaning at ICU discharge.

**Results:**

The primary analysis included 349 intervention patients and 1,768 controls. Successful weaning occurred in 58.3% of intervention patients and 51.7% of controls. In the adjusted primary analysis, the odds ratio for successful weaning was 1.46 (95% CI, 0.99–2.17; *P* = 0.057), and the difference did not reach statistical significance. In prespecified propensity score-matched analysis, successful weaning occurred in 58.3% of intervention patients and 46.4% of matched controls (OR, 2.90; 95% CI, 1.61–5.24; *P* < 0.001). An exploratory analysis restricted to ICU survivors also showed higher odds of successful weaning in the intervention group (OR, 2.10; 95% CI, 1.09–4.04; *P* = 0.026). Overall, 16.3% of intervention patients were transferred to a specialized weaning center. These patients had longer ventilation durations, contributing to a longer overall duration of IMV in the intervention group.

**Conclusions:**

The PRiVENT intervention was feasible and successful weaning was numerically more frequent in the intervention group, although the prespecified adjusted primary analysis did not reach statistical significance. These findings suggest a potential benefit of structured, multidisciplinary weaning support.

**Trial registration:**

ClinicalTrials.gov, NCT05260853, registered on March 2, 2022.

**Supplementary Information:**

The online version contains supplementary material available at https://doi.org/10.1186/s13054-026-06357-3.

## Background

Weaning from invasive mechanical ventilation (IMV) is a pivotal phase of critical illness that strongly influences outcome. Successful weaning is associated with shorter ICU stays and lower mortality, whereas difficult or prolonged weaning is linked to progressively higher mortality [1]. In the multinational WEAN SAFE study, only 65% of patients ventilated for at least two days were successfully weaned by day 90 [2]. Unsuccessful weaning is often followed by long-term invasive ventilation, which is associated with high healthcare utilization, reduced quality of life, and considerable socioeconomic burden [3, 4]. During the early course of IMV, standardized protocols are essential to monitor readiness to wean and to initiate liberation attempts [5]. As weaning becomes difficult or prolonged, management necessitates a comprehensive, multidisciplinary approach to optimize physiological conditions and systematically address the underlying causes of weaning failure in high-risk patients [6, 7]. In Germany, the WeanNet initiative has demonstrated that 64.3% of patients with complex prolonged weaning can be successfully weaned after transfer to specialized weaning centers [8]. However, transfer is often not feasible or cannot be ensured in a timely manner. The duration of IMV prior to transfer is a decisive predictor of outcome since prolonged pre-transfer ventilation is significantly associated with a reduced probability of successful weaning [8]. These observations underscore the need for structured, early-onset strategies that integrate interprofessional expertise directly within the primary ICU. Actively engaging the clinical team at the bedside may mitigate the risks associated with delayed intervention and improve the likelihood of successful weaning. The PRiVENT (PRevention of invasive VENTilation) study was designed to address this gap [9]. Its objective is the early identification of patients at increased risk for prolonged invasive mechanical ventilation and the timely implementation of structured, expert-led, interprofessional interventions to facilitate successful weaning and prevent long-term ventilator dependence, while building sustainable weaning capacity and strengthening interprofessional expertise. In this context, expert-led interprofessional care refers to the integration of specialized weaning expertise, led by pulmonologists with intensive care expertise and respiratory therapists, into the multidisciplinary management of high-risk patients in the primary ICU to identify and address potentially modifiable barriers to successful weaning.

## Methods

### Trial design and oversight

This prospective, multicenter, nonrandomized interventional study involved 40 intensive care units (ICUs) and four DGP-certified specialized weaning centers in Baden-Württemberg, Germany. The intervention cohort, comprising patients at high risk for long-term IMV, was compared with a contemporaneous control cohort derived from statutory health insurance claims data (Allgemeine Ortskrankenkasse, AOK Baden-Württemberg). The study protocol was approved by the ethics committee at each participating site and was previously published [9]. An interim analysis conducted after 12 months, which was restricted to the intervention group, revealed substantial effects of the COVID-19 pandemic on recruitment and study implementation. Therefore, the study period was extended, and the predefined study end date was shifted from June 30, 2023, to December 31, 2024. Written informed consent was obtained from each patient or a legal representative before enrollment. All authors vouch for the accuracy and completeness of the data and for the fidelity of the trial to the protocol.

### Patients

Patients aged ≥ 30 years were screened between 96 h and 11 days after initiation of IMV and were eligible for further risk assessment if they had at least one prespecified pulmonary, cardiovascular, metabolic, malignant, neurologic, psychiatric, or renal comorbidity, or acute COVID-19 pneumonia within this timeframe. Detailed definitions and corresponding ICD-10 codes are provided in Supplementary Appendix S1. Following written informed consent obtained from the patient or, in cases of incapacity, their legally authorized representative, the risk of long-term IMV was assessed using a previously validated prognostic model [10]. Patients classified as being at high risk of weaning failure and all patients receiving IMV for acute COVID-19 pneumonia were assigned to the intervention, as the risk of weaning failure associated with COVID-19 was not yet established and was not captured by the prognostic model. The control cohort comprised AOK Baden-Württemberg-insured patients identified from statutory health-insurance claims data during a largely overlapping contemporary period (Q3/2021–Q3/2024), whereas prospective recruitment into the intervention cohort extended from mid-2021 through the end of 2024. Controls were treated either at hospitals not participating in PRiVENT or, at participating hospitals, before implementation of the intervention at the respective site. Analogous eligibility criteria, including the prespecified ICD-based comorbidity criteria, and the same prognostic model were applied to identify patients at high risk of prolonged IMV in the claims-based control cohort.

In the intervention cohort, patients became eligible for screening after 96 h of IMV. Screening, prognostic risk assessment, informed consent, and enrollment could occur during the subsequent predefined 7-day screening window, with intervention exposure beginning after enrollment. In the claims-based control cohort, eligibility was determined retrospectively by applying analogous eligibility criteria and the same prognostic model. To account for the survival requirement inherent in prospective enrollment, control patients who died within 11 days after initiation of IMV were excluded from the primary analysis set. This 11-day period corresponded to the maximum eligibility period in the intervention cohort, comprising the initial 96 h of IMV and the subsequent 7-day screening window. Correspondingly, intervention patients who died during the screening period before enrollment were excluded. This approach was prespecified to align the eligibility periods between cohorts and minimize potential immortal-time bias. For outcomes defined relative to initiation of IMV, including 30-day mortality and 30-day ventilation duration, follow-up was calculated from the date of initiation of IMV in both cohorts. Thus, although eligibility and intervention exposure were established after initiation of IMV in the intervention cohort, the reference point for these outcomes was initiation of IMV in both groups.

### Intervention

Each of the participating ICUs was assigned to a certified weaning center for consultative support. The PRiVENT intervention comprised a multicomponent, interprofessional weaning program within the primary ICU (Table 1). Following enrollment, risk stratification, and assignment to the intervention group, patient data were transmitted to the affiliated weaning center via an electronic case-report form (eCRF). The core of the intervention was an interdisciplinary Weaning Board convened at the affiliated weaning center, comprising at least one pulmonologist with intensive care certification and one respiratory therapist. Weaning Boards were held at least weekly to provide individualized, pseudonymized treatment recommendations and to plan bedside weaning consultations. This was complemented by interprofessional bedside weaning consultations, with the initial consultation intended to occur within one week after inclusion. The specified frequency referred to the regular provision of Weaning Boards at the weaning-center level; no predefined number of board assessments or bedside consultations per individual patient was required, as repeat assessments were guided by clinical need [11]. This collaborative network also facilitated the timely transfer of high-risk patients to specialized weaning centers when bedside management was deemed insufficient. To enhance institutional expertise, the intervention included e-learning modules developed by the four weaning centers, on-site training sessions, and biannual interprofessional quality circles featuring benchmarking and structured feedback [12]. Public outreach and patient involvement were integrated through digital platforms and cooperation with a charitable organization (https://www.gesundheitstreffpunkt-mannheim.de). Process and health economic evaluations were prespecified; the latter will be reported separately.

**Table 1:**
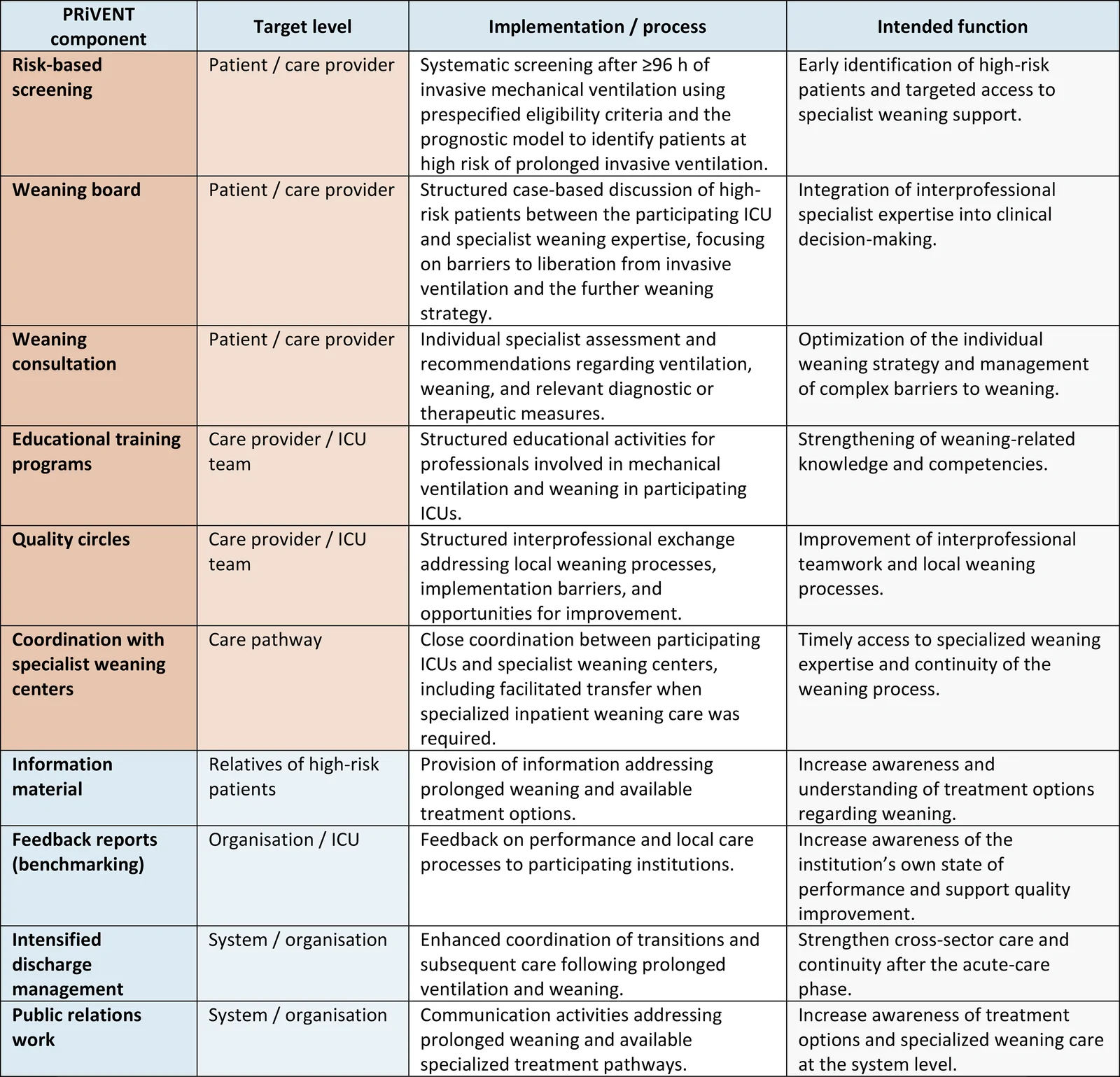
Components and implementation of the multilevel PRiVENT intervention

Note: Core clinical components are highlighted in orange (risk-based screening, weaning board, weaning consultation, and coordination with specialist weaning centers). Supporting implementation components are highlighted in blue (educational training programs, quality circles, information material, feedback reports, intensified discharge management, and public relations work). The components are based on the PRiVENT logic model and are presented here with emphasis on their implementation and intended function within the study

### Primary outcome

The primary outcome was successful weaning from IMV before discharge from the ICU or an affiliated weaning center. Death before successful weaning and an ICU/weaning-center stay exceeding six months after initiation of IMV were classified as weaning failure. Following extension of the study period, the administrative cut-off was extended from June 30, 2023, to December 31, 2024. If the six-month weaning status could not be ascertained because the administrative observation period ended before completion of follow-up, the primary outcome was considered missing and handled using multiple imputation. Outcomes were ascertained through eCRFs for the intervention cohort and statutory health insurance claims data for the control cohort. Because of the different data sources, the primary outcome was operationalized separately in the two cohorts. In the intervention cohort, weaning status was prospectively documented in the eCRF at discharge. In the control cohort, weaning status was derived from claims data using prespecified OPS codes indicating unsuccessful or prolonged weaning. For patients discharged before these codes became mandatory, additional information from directly subsequent hospitalizations and prespecified claims-based indicators of ongoing invasive ventilation was used to determine weaning status (Supplementary Appendix S2).

### Secondary endpoints

Prespecified secondary outcomes included death within 30 days after intubation, in-hospital mortality, duration of mechanical ventilation within 30 days after intubation, and time from intubation to discharge.

### Statistical analysis

The original sample-size calculation assumed approximately 1,495 high-risk patients per group and 90% power to detect a 10-percentage-point absolute increase in successful weaning at a two-sided alpha level of 0.05. Recruitment was substantially affected by the COVID-19 pandemic. A prespecified interim analysis assessed recruitment, screening, data completeness, and the impact of COVID-19 on the enrolled population; descriptive outcome data within the intervention cohort were available, but no comparative intervention-versus-control analyses or estimates of the intervention effect were performed. The study period was subsequently extended by 18 months. A revised calculation anticipated 547 intervention and 2,429 control patients and, after accounting for clustering and dropout, provided 67% power to detect weaning-failure rates of 20% versus 30%. As recruitment remained below projections, propensity-score matching was prospectively added to the statistical analysis plan on September 20, 2023, before comparative intervention-versus-control outcome data were available.

Covariates for the primary model were prespecified a priori in the statistical analysis plan based on clinical relevance and their potential association with weaning outcome; no data-driven statistical variable selection was performed. The model included age, sex, acute COVID-19 pneumonia, Charlson Comorbidity Index, centre experience, pre-existing dependence on ventilatory support for at least three months during the preceding year, new tracheostomy within 96 h after initiation of IMV, and extracorporeal respiratory support (PECLA/ECCO₂R/ECMO or pre-ECMO therapy), with study center included as a random intercept. Prespecified subgroup analyses were performed according to clinically relevant baseline characteristics using the same structure as the primary analysis. The subgroup variable was included both as an independent variable and in interaction with the treatment group variable. The corresponding p-value for the interaction term from a Type III ANOVA was reported to assess potential effect modification. In addition, the primary analysis model was estimated in the subgroups of patients defined by the respective subgroup variables. The propensity-score–matched analysis was performed using all covariates from the primary model, and the variables included in the prognostic model. Propensity scores were estimated using logistic regression (generalized linear model, GLM). Patients were matched 1:1 using nearest-neighbor matching without replacement. No caliper was applied in order to include all intervention patients in the matched analysis. Descriptive characteristics of the matched cohort were given.

Death within 30 days after intubation and in-hospital mortality were analyzed using multivariable mixed-effects logistic-regression models adjusted for the same prespecified covariates as the primary analysis. Duration of mechanical ventilation within 30 days after intubation was analyzed using two prespecified approaches. The adjusted approach incorporated death before day 30 by assigning ventilation time from death until day 30, whereas the unadjusted approach was based solely on observed ventilation hours and did not assign additional ventilation time after death. To ensure data plausibility, ventilation hours exceeding the theoretically possible duration were truncated at the maximum number of hours between intubation and hospital discharge and at 30 days after intubation. Continuous ventilation outcomes were analyzed using mixed-effects linear-regression models adjusted for treatment group, age, sex, acute COVID-19 pneumonia, and Charlson Comorbidity Index, with study center included as a random effect. Time from intubation to discharge was analyzed using a competing-risks approach, with death in the ICU treated as a competing event. The adjusted Fine–Gray subdistribution hazard model included the same prespecified baseline covariates as the primary analysis. Individual patient trajectories in the intervention cohort were additionally assessed to characterize the course of mechanical ventilation and weaning.

## Results

### Patient characteristics

**Fig. 1:**
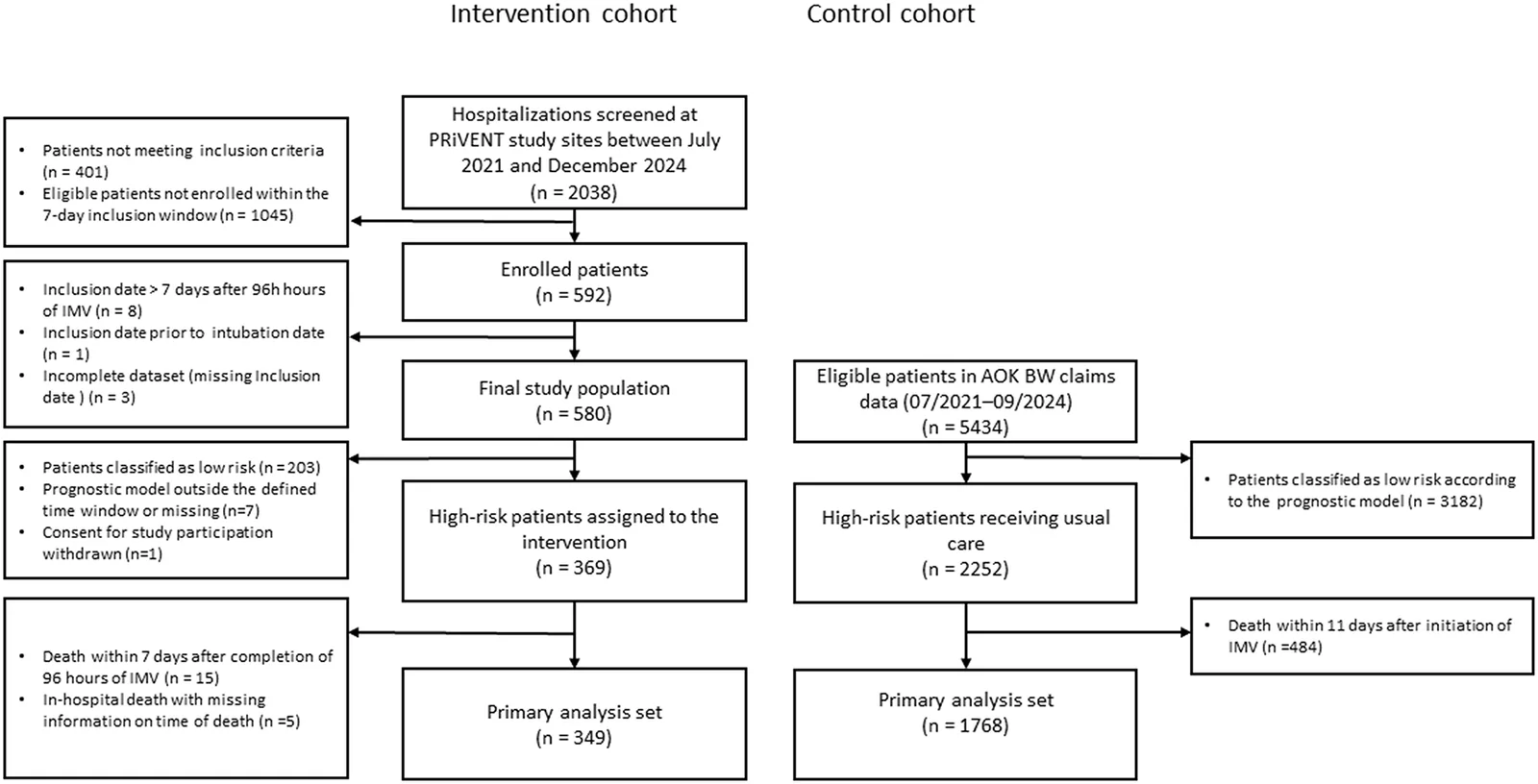
Study population and analysis cohorts. Patients undergoing invasive mechanical ventilation at participating study sites were screened between July 2021 and December 2024. Patients considered to be at high risk for weaning failure and subsequent long-term invasive ventilation according to a prespecified prognostic model were included in the intervention cohort. Control patients were identified from statutory health insurance claims data with the use of the same prognostic model. The figure shows exclusions and the final populations included in the analyses

Between July 2021 and December 2024, a total of 2038 patients were screened at participating ICUs. Recruitment coincided with the third wave of the COVID-19 pandemic and was constrained by hospital access restrictions. Among screened patients in the intervention group, 369 were classified as high risk according to the prespecified prognostic model. After exclusion of 20 patients, 349 patients were included in the primary analysis set. From the claims database we identified 2,252 patients who met all eligibility criteria. Of these, 1,768 survived at least 11 days after intubation and were therefore included in the primary analysis set (Fig. 1). Baseline characteristics are shown in Table 2. The groups were similar with respect to sex and age. However, several preexisting conditions were more prevalent in the control group, including chronic obstructive pulmonary disease (37.1% vs. 30.7%; *P* = 0.03), dementia (4.5% vs. 0.9%; *P* = 0.002), and dependence on a medical suctioning device, mechanical ventilation, or airway secretion management device (20.6% vs. 3.7%; *P* < 0.001). Conversely, the intervention group had higher rates of cardiac arrhythmia (18.6% vs. 0.5%; *P* < 0.001), stroke (10.3% vs. 4.4%; *P* < 0.001), acute pancreatitis (2.9% vs. 0.6%; *P* < 0.001), and dialysis access (2.3% vs. 0.4%; *P* < 0.001). During the initial 96 h after intubation, patients in the intervention group more frequently underwent bronchoscopy (75.1% vs. 45.3%), thoracic or cranial imaging (36.1% vs. 12.7%), surgical procedures involving the cerebrospinal fluid system (4.3% vs. 0.3%), therapeutic positioning (68.2% vs. 4.9%), and plasma transfusion (26.1% vs. 16.1%) (*P* < 0.001 for all comparisons). In contrast, early tracheostomy was more frequent in the control group (34.2% vs. 24.1%; *P* < 0.001).

**Table 2:** Comparison of baseline characteristics in the control and intervention cohorts

Variable	Intervention cohort *N* = 349	Control cohort *N* = 1,768	*P* Value
**Demographic Characteristics**			
Female sex no. (%)	125 (35.82%)	651 (36.82%)	0.768
Age median (IQR), years	63 (55, 71)	67 (59, 74)	0.065
**Clinical and Care-Dependency Characteristics — no. (%)**			
Nursing home residence before admission	10 (2.87%)	41 (2.32%)	0.676
Hospitalization > 21 days *	20 (5.73%)	148 (8.37%)	0.119
Preexisting invasive home mechanical ventilation*	5 (1.43%)	12 (0.68%)	0.265
Dependence on home suction and/or mechanical ventilation or airway secretion management devices.**	13 (3.72%)	365 (20.64%)	**< 0.001**
Permanent or temporary tracheostomy*	11 (3.15%)	32 (1.81%)	0.157
Dialysis fistula, shunt, or bypass*	8 (2.29%)	7 (0.40%)	**< 0.001**
**COVID-19 Status — no. (%)**			
Acute COVID-19 pneumonia	95 (27.22%)	573 (32.41%)	0.065
**Selected admission diagnoses — no. (%)** ^**§**^			
Cardiac arrhythmia	65 (18.62%)	9 (0.51%)	**< 0.001**
Stroke	36 (10.32%)	77 (4.36%)	**< 0.001**
Acute pancreatitis	10 (2.87%)	11 (0.62%)	**< 0.001**
**Coexisting conditions — no. (%)**			
Thyroiditis	9 (2.58%)	53 (3.0%)	0.802
Dementia	3 (0.86%)	79 (4.47%)	0.002
Eating disorder	26 (7.45%)	22 (1.24%)	**< 0.001**
Rheumatic mitral valve disease (stenosis or insufficiency)	3 (0.86%)	42 (2.38%)	0.112
Peritonitis	7 (2.01%)	13 (0.74%)	0.052
Pneumothorax	18 (5.16%)	39 (2.21%)	**0.003**
Obesity (BMI > 35 kg/m²)	120 (34.38%)	709 (40.10%)	0.052
Chronic obstructive pulmonary disease	107 (30.66%)	655 (37.05%)	0.027
Pulmonary or abdominal metastases	5 (1.43%)	26 (1.47%)	> 0.999
**Charlson Comorbidity Score — median (IQR)**			
Charlson comorbidity score	3.00 (1.00, 4.00)	5.00 (3.00, 7.00)	**< 0.001**
**Procedures during the current ICU stay (≤ 95 h after intubation) — no. (%)**			
Bronchoscopy	262 (75.07%)	801 (45.31%)	< 0.001
Non–contrast-enhanced chest CT	239 (68.48%)	259 (14.65%)	**< 0.001**
Contrast-enhanced cranial CT and/or MRI	126 (36.10%)	224 (12.67%)	**< 0.001**
Surgical procedures involving the cerebrospinal fluid system (e.g., drainage, shunt, or catheter placement or removal)	15 (4.30%)	5 (0.28%)	**< 0.001**
Tracheostomy	84 (24.07%)	605 (34.22%)	**< 0.001**
Neck dissection	0 (0.0%)	13 (0.74%)	0.218
Chest-tube placement	59 (16.91%)	241 (13.63%)	0.129
Therapeutic positioning in a specialized bed (e.g., rotating or sandwich bed)	238 (68.19%)	86 (4.86%)	**< 0.001**
Autologous blood collection and transfusion	3 (0.86%)	45 (2.55%)	0.082
Transfusion of plasma components or recombinant plasma proteins	91 (26.07%)	285 (16.12%)	**< 0.001**
Extracorporeal respiratory support (PECLA, ECCO₂R, veno–venous or veno–arterial ECMO), including pre-ECMO therapy	35 (10.03%)	138 (7.81%)	0.201
Complex treatment for colonization or infection with multidrug-resistant pathogens	20 (5.73%)	51 (2.88%)	**0.011**

The variables shown correspond to patient characteristics that were identified as independently associated with the primary end point in previous epidemiological analyses used for the development of the prognostic model. These associations were derived from the original AOK training cohort (2015–2017) and subsequently confirmed in an external validation dataset (2018). In addition, COVID-19 status was included as an additional variable, as it was not available in the earlier study periods [10]

* Year preceding the index hospitalization

** More than 3 months during the preceding year

### Implementation and process evaluation

503 Weaning Board assessments were conducted in 83.6% of intervention patients, with a mean of 1.44 and a median of 1 (IQR, 1–2) assessments per patient. Overall, 390 bedside weaning consultations were conducted in 62.2% of patients, with a mean of 1.12 and a median of 1 (IQR, 0–2) consultations per patient. In addition, 214 health-care professionals participated in the e-learning programme.

### Primary outcome

**Fig. 2:**
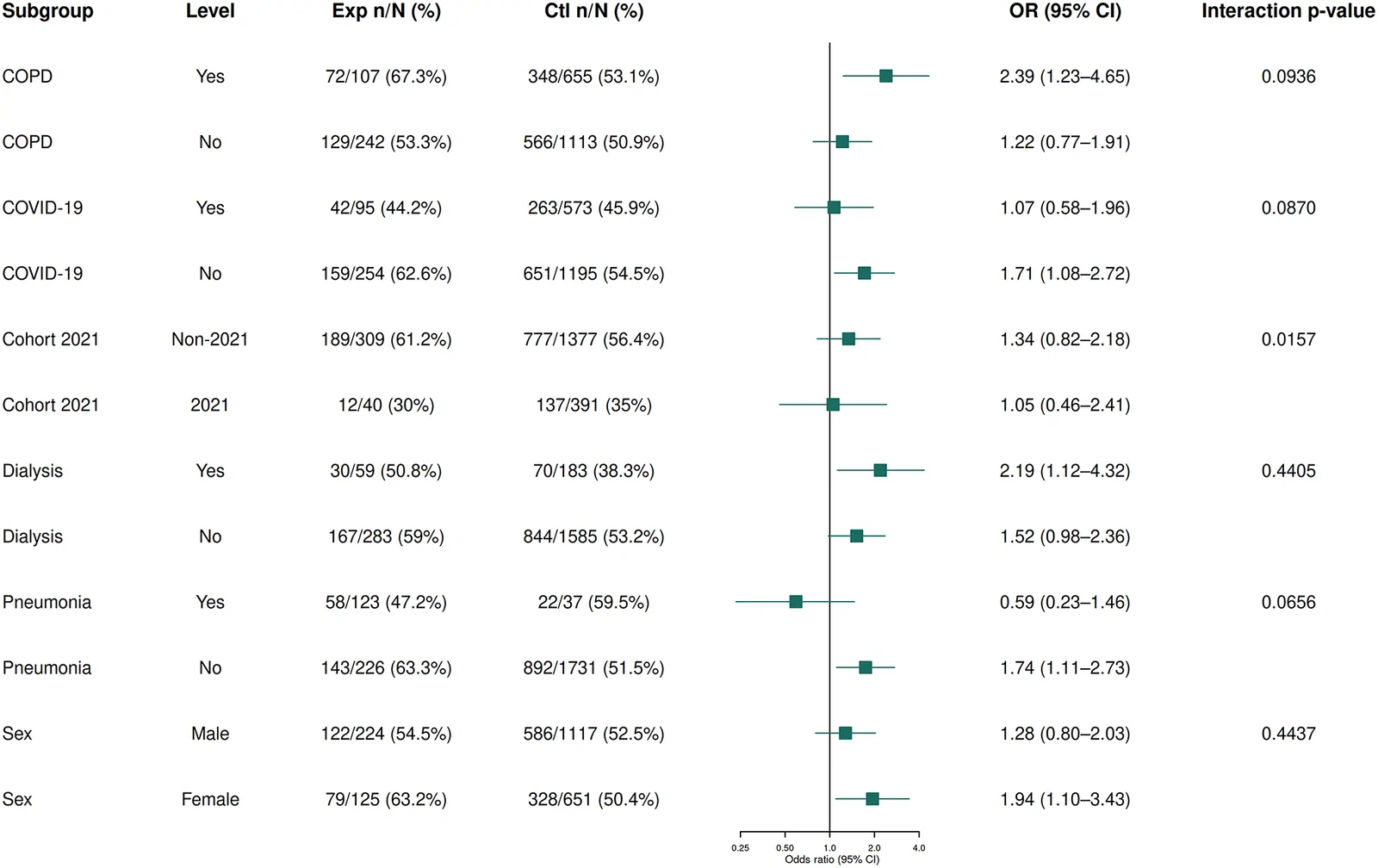
Subgroup analyses of successful weaning at ICU discharge. Forest plot showing odds ratios and 95% confidence intervals for successful weaning in predefined patient subgroups. Odds ratios greater than 1 indicate a higher likelihood of successful weaning in the intervention group than in the control group

Successful weaning occurred in 201 of 349 patients (58.3%; 4 missing values) in the intervention group and in 914 of 1768 patients (51.7%) in the control group (*P* = 0.03). In the prespecified adjusted mixed-effects logistic-regression analysis, the odds ratio for successful weaning was 1.46 (95% CI, 0.99–2.17; *P* = 0.057), and the difference did not reach statistical significance. Baseline characteristics of the propensity score-matched cohort are shown in Supplementary Table S2. In the prespecified propensity-score–matched analysis, which included 349 matched pairs, successful weaning occurred in 58.3% of patients in the intervention group and in 46.4% of patients in the control group (Supplementary Table S3). The adjusted odds ratio for successful weaning was 2.90 (95% CI, 1.61 to 5.24; *P* = 0.0004). Primary and secondary outcomes are shown in Table 3. In an exploratory analysis restricted to ICU survivors, successful weaning was more frequent in the intervention group than in the control group (OR, 2.10; 95% CI, 1.09–4.04; *P* = 0.026). Subgroup-specific point estimates generally favored the intervention, although effect estimates varied across subgroups. The strongest associations with successful weaning were observed in patients with COPD (OR 2.39; 95% CI 1.23–4.65), female patients (OR 1.94; 95% CI 1.10–3.43), patients requiring dialysis (OR 2.19; 95% CI 1.12–4.32), and patients without COVID-19 at screening (OR 1.71; 95% CI 1.08–2.72). Similarly, patients without pneumonia at screening showed improved weaning success (OR 1.74; 95% CI 1.11–2.73). Point estimates were closer to the null in patients with pneumonia or COVID-19 pneumonia. Formal interaction tests were performed for all predefined subgroup variables; corresponding interaction P values are shown in Fig. 2.

**Table 3:** Primary and secondary outcomes

Outcome	*N*	Intervention *N* (%); Median (Q1, Q3)	Control *N* (%); Median (Q1, Q3)	Effect estimate (95% CI)	*P* Value
**Primary outcome (Successful Weaning)**					
Primary analysis (full cohort)	2,117	201 (58.3%)	914 (51.7%)	1.46 (0.99–2.17) ‡	0.057
Propensity-score–matched analysis*	698	201 (58.3%)	162 (46.42%)	2.90 (1.61–5.24) ‡	< 0.001
Subgroup of ICU survivors	1540	201 (78%)	913 (71%)	2.10 (1.09–4.04) ‡	0.026
**Secondary Outcomes**					
Death within 30 days of intubation	2,114	63 (18.21%)	427 (24.15%)	0.78 (0.55–1.11)	0.17
In-hospital mortality**	2,116	88 (25.29%)	484 (27.38%)	0.93(0.64–1.35) ‡	0.70
30-day MV hours, Unadjusted Approach	2,117	456 (299, 700)	342 (206,547)	81 (49–114) ‡‡	< 0.001
30-day MV hours, Adjusted Approach	2,117	576 (316, 720)	449 (227, 718)	74 (41–107) ‡‡	< 0.001
Total MV duration during the index hospitalization	2,117	408 (288,600)	342 (206, 547)	67 (11, 124) ‡‡	0.020

† P value from chi-square test comparing proportions between groups. ‡ Adjusted ORs were estimated with a mixed-effects logistic regression model adjusted for age, sex, COVID-19 pneumonia, Charlson Comorbidity Index, prior Dependence on home suction and/or mechanical ventilation or airway secretion management devices, early tracheostomy, ECMO, and center experience; center was included as a random intercept

‡‡ ‡‡ β, estimated with a mixed-effects linear regression model adjusted for treatment group, age, sex, acute COVID-19 pneumonia, and Charlson Comorbidity Index (continuous), with center included as a random effect

* Based on 349 matched pairs. One patient was excluded from the secondary endpoint analyses due to missing follow-up data after transfer to a specialized weaning center; no imputation was performed. Unadjusted approach, only observed ventilation hours were considered. ***Adjusted approach, patients who died before day 30 were assigned ventilation time until day 30

### Secondary outcomes

**Fig. 3:**
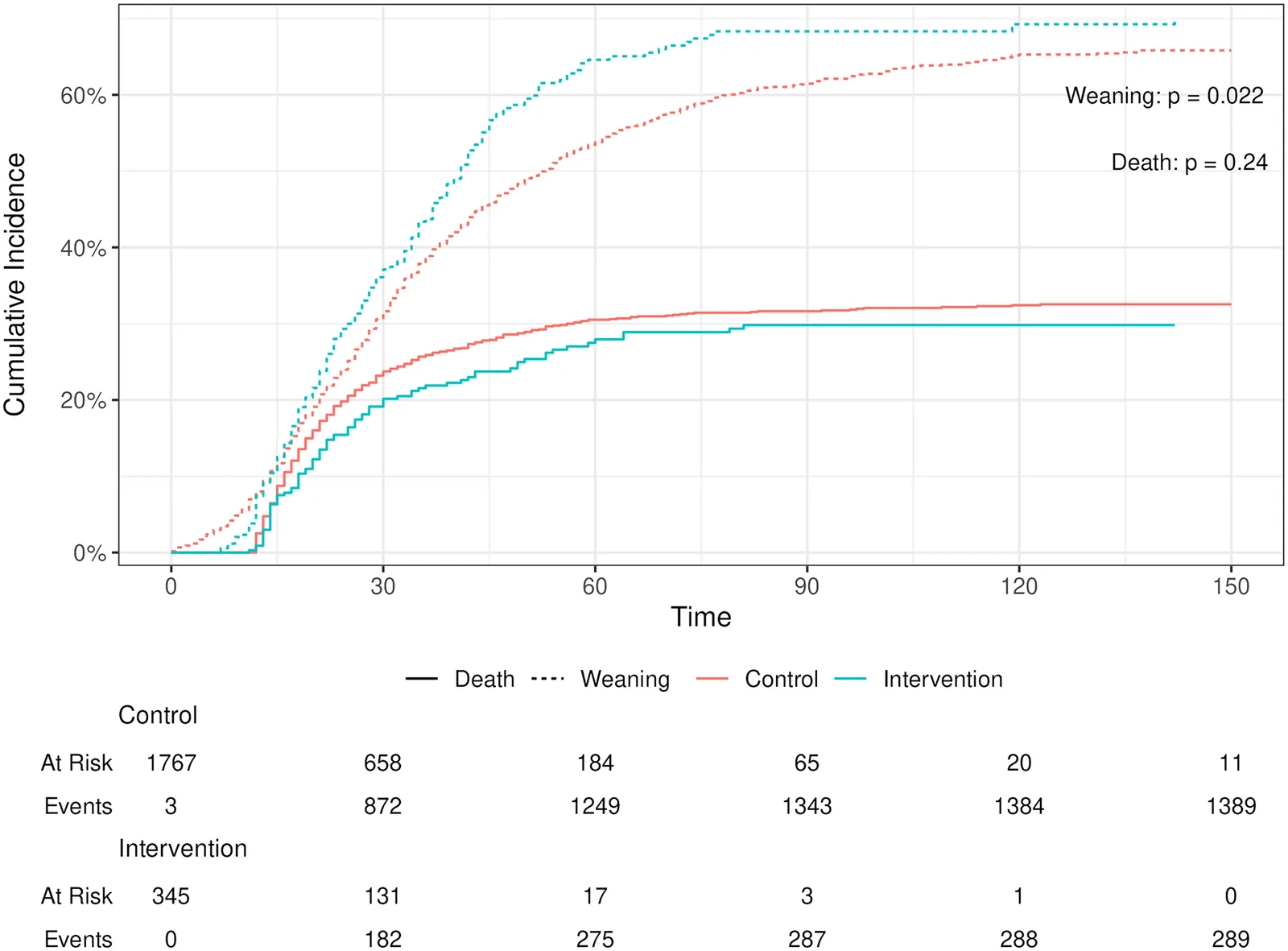
Cumulative Incidence of Successful Weaning and Death According to Study Group. Cumulative-incidence curves for successful weaning and death from the time of intubation in the intervention group (exp) and control group (ctl), accounting for competing risks. Blue curves indicate successful weaning; red curves indicate death. Solid lines represent the intervention group, and dashed lines the control group. The cumulative incidence of successful weaning differed significantly between groups (Gray’s test, *P* = 0.02), whereas cumulative incidence of death did not differ significantly (*P* = 0.24)

There was no statistically significant difference in mortality outcomes between the two groups. Death within 30 days after intubation occurred in 18.2% of patients in the intervention group and 24.2% of those in the control group (adjusted odds ratio, 0.78; 95% confidence interval [CI], 0.55 to 1.11; *P* = 0.17). In-hospital mortality was 25.3% and 27.4%, respectively (adjusted odds ratio, 0.93; 95% CI, 0.64 to 1.35; *P* = 0.70). Patients in the intervention group accumulated more hours of mechanical ventilation than those in the control group. In the unadjusted analysis, in which only observed ventilation hours were considered, the median duration of mechanical ventilation within 30 days after intubation was 456 h (interquartile range [IQR], 299 to 700) in the intervention group and 342 h (IQR, 206 to 547) in the control group, corresponding to an adjusted increase of 81 h (95% CI, 49 to 114; *P* < 0.001). Similar findings were observed in the adjusted analysis, in which patients who died before day 30 were assigned ventilation time until day 30 (adjusted increase, 74 h; 95% CI, 41 to 107; *P* < 0.001). The total duration of mechanical ventilation during the index hospitalization was also longer in the intervention group than in the control group (median, 408 h [IQR, 288 to 600] vs. 342 h [IQR, 206 to 547]; adjusted increase, 67 h; 95% CI, 11 to 124; *P* = 0.02), see Table 3. Transfer to a specialized weaning center occurred in 57 of 349 patients (16.3%) in the intervention group and in none of the controls. Intervention patients transferred to a weaning center accumulated substantially more ventilation hours than those managed without transfer (median, 791 h [IQR, 432 to 1007] vs. 384 h [IQR, 275 to 549]). Swimmer plots showed heterogeneous clinical trajectories within the intervention cohort, including prolonged mechanical ventilation among patients with unsuccessful weaning and those transferred to specialized weaning centers (Supplementary Figure S1). Successful weaning from long-term mechanical ventilation was confirmed at hospital discharge. Since patients could die before successful weaning could be achieved, death was considered a competing event for this endpoint. The cumulative incidence of successful weaning by the time of hospital discharge differed significantly between the treatment groups (Gray test: *p* = 0.022). The intervention group showed a higher cumulative incidence of successful weaning over the observation period compared to the control group. In contrast, no statistically significant difference was observed between the groups for the competing event of death (Gray test: *p* = 0.238). In the Fine-Gray model (adjusted for the variables of the primary model), no significant difference was observed between the groups regarding the cumulative probability of weaning (sHR 1.12; 95% CI: 0.91–1.38; *p* = 0.30) or the probability of death (sHR 0.87; 95% CI 0.67–1.11; *p* = 0.26). Accounting for death as a competing event provides a complementary assessment of time to successful weaning while avoiding treatment of death as non-informative censoring (Fig. 3).

## Discussion

In this prospective, multicenter, nonrandomized study, successful weaning was numerically more frequent among patients receiving the PRiVENT intervention than among controls, although the prespecified adjusted primary analysis did not reach conventional statistical significance. Owing to the COVID-19 pandemic and its impact on ICU resources and study recruitment, substantially fewer patients were enrolled than originally planned. Consequently, the study may have been underpowered to demonstrate a statistically significant treatment effect in the overall cohort. Nevertheless, the point estimate of the adjusted primary analysis was above 1, while the prespecified propensity score-matched analysis yielded a larger effect estimate. Successful weaning was also more frequent in the intervention group in the analysis restricted to ICU survivors. However, because survival is a post-intervention variable, this analysis should be considered exploratory and interpreted cautiously due to the potential for selection bias. The overall weaning success rate of 58.3% in the intervention group underscores the severity of illness and the high risk of prolonged weaning and long-term invasive mechanical ventilation in this population. Consistent with findings from the WIND and WEAN SAFE studies [1, 2], the early phase after initiation of invasive mechanical ventilation appears to represent a critical window for intervention. The PRiVENT intervention was designed to support weaning during this period through structured interprofessional boards, coordinated involvement of specialized weaning expertise, and targeted knowledge transfer involving all members of the ICU care team. Initial results from the accompanying healthcare professional surveys indicate that staff participating in PRiVENT are generally satisfied with their working conditions and perceive interprofessional collaboration as good. Ongoing follow-up surveys will further evaluate whether the intervention contributes to sustained improvements in interprofessional cooperation, communication, and job satisfaction among healthcare professionals [13].

The competing-risk analysis provides a complementary perspective on the primary findings. Although the unadjusted cumulative incidence of successful weaning differed between groups, this association was no longer statistically significant after covariate adjustment in the Fine–Gray model. Unlike the binary primary analysis, the Fine–Gray model accounts for both the timing of successful weaning and death as a competing event. Mechanical ventilation duration was significantly longer in the intervention group. Patients transferred to specialized weaning centers had particularly long ventilation durations; however, the observational data do not allow us to determine whether transfer, prolonged weaning attempts, differences in patient characteristics, or other factors accounted for this finding. Accordingly, the longer duration of mechanical ventilation should not be interpreted as either beneficial or detrimental based on the present analysis. A substantial number of eligible patients were not enrolled within the predefined inclusion window, raising the possibility of selective recruitment of patients perceived as most likely to benefit from the intervention. While standardized protocols for spontaneous breathing trials may reduce ventilation duration, difficult and prolonged weaning are typically multifactorial and often require an individualized and comprehensive treatment approach [14, 15]. This ‘peeling off’ of supportive layers—including optimized nutrition, psychological support, and specialized nursing, is traditionally reserved for specialized weaning centers or long-term acute care facilities [16]. The PRiVENT study operationalized this multidisciplinary model within the acute ICU setting. Unlike previous investigations of weaning intervention bundles or quality improvement programs that focus on early protocol-directed weaning and specialized surgical cohorts, our study transplanted expert-level multidisciplinary coordination into a larger, non-specialized ICU network [17, 18]. While small-scale audits and studies in weaning centers previously suggested benefits of such teams, PRiVENT bridges the gap between acute intensive care and specialized weaning expertise at a much earlier stage of the weaning process [19, 20]. The clinical relevance of the observed difference in weaning success must also be considered in relation to the resources required to implement the multicomponent intervention. Delivery required regular specialist Weaning Boards, bedside consultations, educational activities, and coordination between participating ICUs and specialized weaning centers. Given the uncertainty of the primary effect estimate, the present findings therefore do not establish that the potential clinical benefit outweighs the additional organizational and personnel requirements. Further evaluation of implementation, resource use, and cost-effectiveness is needed before broader adoption can be recommended.

## Limitations

A major limitation of this study is its nonrandomized design. A cluster-randomized controlled trial with randomization at the ICU level would have provided a more robust assessment of the intervention effect while minimizing contamination between treatment groups. Residual confounding and selection bias therefore cannot be excluded despite multivariable adjustment and propensity-score matching. In addition, different data sources were used for the two cohorts. Although the same conceptual primary outcome was applied, weaning status was prospectively documented in the intervention cohort but reconstructed from prespecified claims-based indicators in controls, introducing the possibility of differential outcome ascertainment and misclassification. Implementation of individual intervention components varied, and bedside weaning consultations were not performed in all intervention patients; available process data do not distinguish clinically intended non-delivery from incomplete implementation. Finally, recruitment was substantially affected by the COVID-19 pandemic. Despite an 18-month extension, only 349 intervention patients were included in the primary analysis set, resulting in lower statistical power than originally planned. Approximately one quarter of the intervention cohort had COVID-19 pneumonia; subgroup estimates were imprecise, and and formal interaction testing provided no clear evidence of effect modification.

## Conclusion

PRiVENT demonstrates the feasibility of implementing structured, multidisciplinary weaning support across a regional network of ICUs and specialized weaning centers. Successful weaning was numerically more frequent in the intervention group, but the prespecified adjusted primary analysis did not reach statistical significance. Accordingly, the findings suggest a potential benefit, while uncertainty regarding the magnitude and causal nature of the association remains.

## Supplementary Information

Below is the link to the electronic supplementary material.

Supplementary Material 1

## Data Availability

No datasets were generated or analysed during the current study.

## References

[1] Beduneau G, Pham T, Schortgen F, Piquilloud L, Zogheib E, Jonas M, et al. Epidemiology of Weaning Outcome according to a New Definition. The WIND Study. Am J Respir Crit Care Med. 2017;195(6):772–83. 10.1164/rccm.201602-0320OC 27626706

[2] Pham T, Heunks L, Bellani G, Madotto F, Aragao I, Beduneau G, et al. Weaning from mechanical ventilation in intensive care units across 50 countries (WEAN SAFE): a multicentre, prospective, observational cohort study. Lancet Respir Med. 2023;11(5):465–76. 10.1016/S2213-2600(22)00449-0 36693401

[3] Huttmann SE, Magnet FS, Karagiannidis C, Storre JH, Windisch W. Quality of life and life satisfaction are severely impaired in patients with long-term invasive ventilation following ICU treatment and unsuccessful weaning. Ann Intensive Care. 2018;8(1):38. 10.1186/s13613-018-0384-8 PMC5856853 29549456

[4] Michels JD, Trudzinski FC, Bornitz F, Ewert R, Muller M, Trinkmann F, et al. Costs of weaning failure: a prospective, multicentre, controlled, non-randomised, interventional study on economic implications for the German health care system. respiration. 2023;102(9):813–20. 10.1159/000533333 37619539

[5] Girard TD, Alhazzani W, Kress JP, Ouellette DR, Schmidt GA, Truwit JD, et al. An Official American Thoracic Society/American College of Chest Physicians Clinical Practice Guideline: Liberation from Mechanical Ventilation in Critically Ill Adults. Rehabilitation Protocols, Ventilator Liberation Protocols, and Cuff Leak Tests. Am J Respir Crit Care Med. 2017;195(1):120–33. 10.1164/rccm.201610-2075ST 27762595

[6] Shah NM, Hart N, Kaltsakas G. Prolonged weaning from mechanical ventilation: who, what, when and how? Breathe (Sheff). 2024;20(3):240122. 10.1183/20734735.0122-2024 PMC11629167 39660085

[7] Heunks LM, van der Hoeven JG. Clinical review: the ABC of weaning failure–a structured approach. Crit Care. 2010;14(6):245. 10.1186/cc9296 PMC3220047 21143773

[8] Windisch W, Dellweg D, Geiseler J, Westhoff M, Pfeifer M, Suchi S, et al. Prolonged Weaning from Mechanical Ventilation. Dtsch Arztebl Int. 2020;117(12):197–204. 10.3238/arztebl.2020.0197 PMC7194302 32343653

[9] Michels JD, Meis J, Sturm N, Bornitz F, von Schumann S, Weis A, et al. Prevention of invasive ventilation (PRiVENT)-a prospective, mixed-methods interventional, multicentre study with a parallel comparison group: study protocol. BMC Health Serv Res. 2023;23(1):305. 10.1186/s12913-023-09283-0 PMC10061400 36998047

[10] Trudzinski FC, Michels-Zetsche JD, Neetz B, Meis J, Muller M, Kempa A, et al. Risk factors for long-term invasive mechanical ventilation: a longitudinal study using German health claims data. Respir Res. 2024;25(1):60. 10.1186/s12931-024-02693-6 PMC10821552 38281006

[11] Keller S, Forstner J, Weis A, Biehler E, Trudzinski F, Michels J, et al. Interprofessional weaning boards and weaning consults for long-term ventilated patients: A qualitative study of perceived potential for patient care. Pneumologie. 2024;78(3):180–90. 10.1055/a-2161-6014 37857322

[12] Michels-Zetsche JD, Schubert-Haack J, Tanck K, Neetz B, Iberl G, Muller M, et al. E-learning-an interventional element of the PRiVENT project to improve weaning expertise. BMC Med Educ. 2024;24(1):420. 10.1186/s12909-024-05416-z PMC11027525 38641835

[13] Fleischhauer T, Biehler E, Michels-Zetsche JD, Trudzinski FC, Schubert-Haack J, Forstner J, et al. Interprofessional Collaboration and Job Satisfaction in German Intensive Care Units: A Cross-Sectional Survey within the PRiVENT Project. Respiration. 2025;104(10):761–8. 10.1159/000546043 PMC12185057 40418908

[14] Trudzinski FC, Neetz B, Dahlhoff JC, Wilkens FM, Katzenschlager S, Fahndrich S, et al. A multidimensional approach to the management of patients in prolonged weaning from mechanical ventilation: the concept of treatable traits - a narrative review. Respiration. 2025;104(4):240–54. 10.1159/000541965 39476811

[15] Trudzinski FC, Neetz B, Bornitz F, Muller M, Weis A, Kronsteiner D, et al. Risk factors for prolonged mechanical ventilation and eaning failure: a systematic review. Respiration. 2022;101(10):959–69. 10.1159/000525604 PMC9677859 35977525

[16] White AC. Long-term mechanical ventilation: management strategies. Respir Care. 2012;57(6):889–97. discussion 98 – 9. 10.4187/respcare.01850 22663965

[17] Sterr F, Bauernfeind L, Knop M, Rester C, Metzing S, Palm R. Weaning-associated interventions for ventilated intensive care patients: A scoping review. Nurs Crit Care. 2024;29(6):1564–79. 10.1111/nicc.13143 39155350

[18] Hefner JL, Tripathi RS, Abel EE, Farneman M, Galloway J, Moffatt-Bruce SD. Quality Improvement Intervention to Decrease Prolonged Mechanical Ventilation After Coronary Artery Bypass Surgery. Am J Crit Care. 2016;25(5):423–30. 10.4037/ajcc2016165 27587423

[19] Black CJ, Kuper M, Bellingan GJ, Batson S, Matejowsky C, Howell DC. A multidisciplinary team approach to weaning from prolonged mechanical ventilation. Br J Hosp Med (Lond). 2012;73(8):462–6. 10.12968/hmed.2012.73.8.462 22875526

[20] Clini EM, Crisafulli E, Antoni FD, Beneventi C, Trianni L, Costi S, et al. Functional recovery following physical training in tracheotomized and chronically ventilated patients. Respir Care. 2011;56(3):306–13. 10.4187/respcare.00956 21235844

[21] Zhu B, Li Z, Jiang L, Du B, Jiang Q, Wang M, et al. Effect of a quality improvement program on weaning from mechanical ventilation: a cluster randomized trial. Intensive Care Med. 2015;41(10):1781–90. 10.1007/s00134-015-3958-z 26156108

