# Supplementary material for: A specialist-led interprofessional weaning program to prevent weaning failure in high-risk patients: the PRiVENT study
Source: Crit Care. 2026 Sep 30;30:507. doi: 10.1186/s13054-026-06357-3 (PMC13628980; doi:10.1186/s13054-026-06357-3)
Supplement: Supplementary file 1 [file 13054_2026_6357_MOESM1_ESM.docx]

## 1. Supplementary Appendix S1. ICD-10 codes used to define prespecified comorbidities for eligibility screening

| **Prespecified condition** | **ICD-10 code(s)** |
| --- | --- |
| Chronic obstructive pulmonary disease (COPD) | J44 |
| Scoliosis | M41 |
| Other chronic lung diseases | J60–J70 |
| Interstitial lung disease | J84 |
| Heart failure | I50 |
| Chronic ischemic heart disease | I25 |
| Diabetes mellitus | E10–E14 |
| Obesity | E66.01, E66.02 |
| Malignancy | C00–C97 |
| Delirium, including substance-related withdrawal delirium | F05; F10.4–F16.4; F17.4; F18.4; F19.4 |
| Schizophrenia, schizotypal, and delusional disorders | F20–F29 |
| Critical illness polyneuropathy/myopathy (CIP/CIM) | G62.80, G72.80 |
| Acute kidney injury | N17 |
| Chronic kidney disease | N18 |

**Table S1. ICD-10 codes used to define prespecified comorbidities for eligibility screening**

**Exclusion criterion:** Patients with neuromuscular diseases considered to substantially affect weaning potential were excluded (ICD-10 codes G12.2 and/or G71).

## 2. Supplementary Appendix S2. Determination of ventilator dependence in the control cohort

Ventilator dependence at ICU discharge or hospital discharge was determined according to the following hierarchical algorithm:

1. **Death before discharge from index hospitalization** → Classified as weaning failure.
2. More than 6 months between intubation and discharge from index hospitalisation

🡪 classified as weaning failure

3. Presence of OPS code **1-717.0** or **1-717.1** at discharge or transfer
→ Classified as ongoing invasive mechanical ventilation (weaning failure).

3. **Cases with discharge date before January 1, 2022**
   Ventilator dependence was assumed if any of the following criteria were met:
   - Discharge with durable medical equipment code **12.50.99.0002** (home invasive ventilation), or
   - Documentation of OPS codes **8-7601**, **8-7611**, or **8-7621** indicating prolonged invasive ventilation without subsequent documentation of successful weaning, or
   - Concurrent ICD-10-GM codes **Z99.1** and **Z43.0** at discharge, or
   - Chained hospitalizations indicating uninterrupted invasive mechanical ventilation.
4. **No criteria met**
   → Classified as successfully weaned.

Follow-up extended from the date of intubation to 30 days after discharge from the index hospitalization or until death, whichever occurred first.

## Rationale for Code Selection

Beginning January 1, 2022, OPS codes **1-717.0** and **1-717.1** were introduced as mandatory procedure codes to specifically document prolonged weaning and continued invasive mechanical ventilation at discharge or transfer. Because these codes explicitly reflect structured weaning processes and ventilator status, they were used as the primary markers of ventilator dependence for cases from 2022 onward.

For earlier cases, no dedicated OPS code for prolonged weaning was available. Therefore, ventilator dependence was ascertained using a prespecified combination of procedure codes, durable medical equipment codes, diagnosis codes, and hospitalization patterns. The combined use of these indicators was intended to maximize sensitivity for detecting ongoing invasive mechanical ventilation while maintaining specificity for clinically relevant ventilator dependence at discharge.

## 3. Supplementary Table S*2*. Baseline characteristics of the propensity score-matched cohort

| Variable | Overall  N = 698 | Intervention group N = 349 | Control group N = 349 | P value* |
| --- | --- | --- | --- | --- |
| Acute COVID-19 pneumonia |  |  |  | >0,999 |
| Yes | 191 (27%) | 95 (27%) | 96 (28%) |  |
| No | 507 (73%) | 254 (73%) | 253 (72%) |  |
| Age |  |  |  | 0,093 |
| Mean ± SD | 63,11 ± 11,93 | 62,36 ± 11,96 | 63,87 ± 11,86 |  |
| Median (Q1, Q3) | 63,00  (56,00; 72,00) | 63,00  (55,00; 71,00) | 64,00  (57,00; 72,00) |  |
| Min, Max | 30,00; 90,00 | 30,00; 88,00 | 31,00; 90,00 |  |
| Sex |  |  |  | 0,349 |
| Male | 435(62%) | 224(64%) | 211(60%) |  |
| Female | 263(38%) | 125(36%) | 138(40%) |  |
| Charlson comorbidity score |  |  |  | <0,001 |
| Mean ± SD | 3,51 ± 2,31 | 3,05 ± 2,30 | 3,97 ± 2,23 |  |
| Median (Q1, Q3) | 3,00 (2,00; 5,00) | 3,00 (1,00; 4,00) | 4,00 (2,00; 5,00) |  |
| Min, Max | 0,00; 12,00 | 0,00; 11,00 | 0,00; 12,00 |  |
| Center experience |  |  |  | 0,003 |
| Less experienced | 290 (42%) | 125 (36%) | 165 (47%) |  |
| Experienced | 408 (58%) | 224 (64%) | 184 (53%) |  |
| Dependence on home mechanical ventilation and/or airway secretion management |  |  |  | 0,002 |
| Yes | 48 (7%) | 13 (4%) | 35 (10%) |  |
| No | 650 (93%) | 336 (96%) | 314 (90%) |  |
| Akute Tracheostomie |  |  |  | 0,019 |
| Yes | 197 (28%) | 84 (24%) | 113 (32%) |  |
| No | 501 (72%) | 265 (76%) | 236 (68%) |  |
| Extracorporeal membrane oxygenation |  |  |  | 0,014 |
| Yes | 52 (7%) | 35 (10%) | 17 (5%) |  |
| No | 646 (93%) | 314 (90%) | 332 (95%) |  |

Baseline characteristics of the 1:1 propensity score-matched per-protocol analysis set (PAS), stratified by study group

*Pearson-Chi-Quadrat-Test; Welch-Zweistichproben-t-Test

## 4. Supplementary Table S3. Primary outcome and ICU mortality in the propensity score-matched cohort

| Variable | N | Gesamt N = 698* | IG N = 349 | KG N = 349 | p-Wert* |
| --- | --- | --- | --- | --- | --- |
| Successful weaning | 694 |  |  |  | 0,002 |
| Yes |  | 363 (52%) | 201 (58%) | 162 (46%) |  |
| No |  | 331 (48%) | 144 (42%) | 187 (54%) |  |
| ICU mortality | 697 |  |  |  | 0,563 |
| Yes |  | 184 (26%) | 88 (25%) | 96 (28%) |  |
| No |  | 513 (74%) | 260 (75%) | 253 (72%) |  |

*Pearson-Chi-Quadrat-Test; Welch-Zweistichproben-t-Test*.* Four primary-outcome values were missing in the intervention group and are therefore not included in the descriptive counts presented here; these missing values were handled by multiple imputation in the primary outcome analysis.

## 5. Supplementary Figure S1. Individual trajectories of mechanical ventilation and weaning
